# Supplementary material for: An IgY Effectively Prevents Goslings from Virulent GAstV Infection
Source: Vaccines (Basel). 2022 Dec 7;10(12):2090. doi: 10.3390/vaccines10122090 (PMC9781778; doi:10.3390/vaccines10122090)
Supplement: Supplementary file 1 [file vaccines-10-02090-s001.zip › vaccines-2045265-supplementary.pdf]

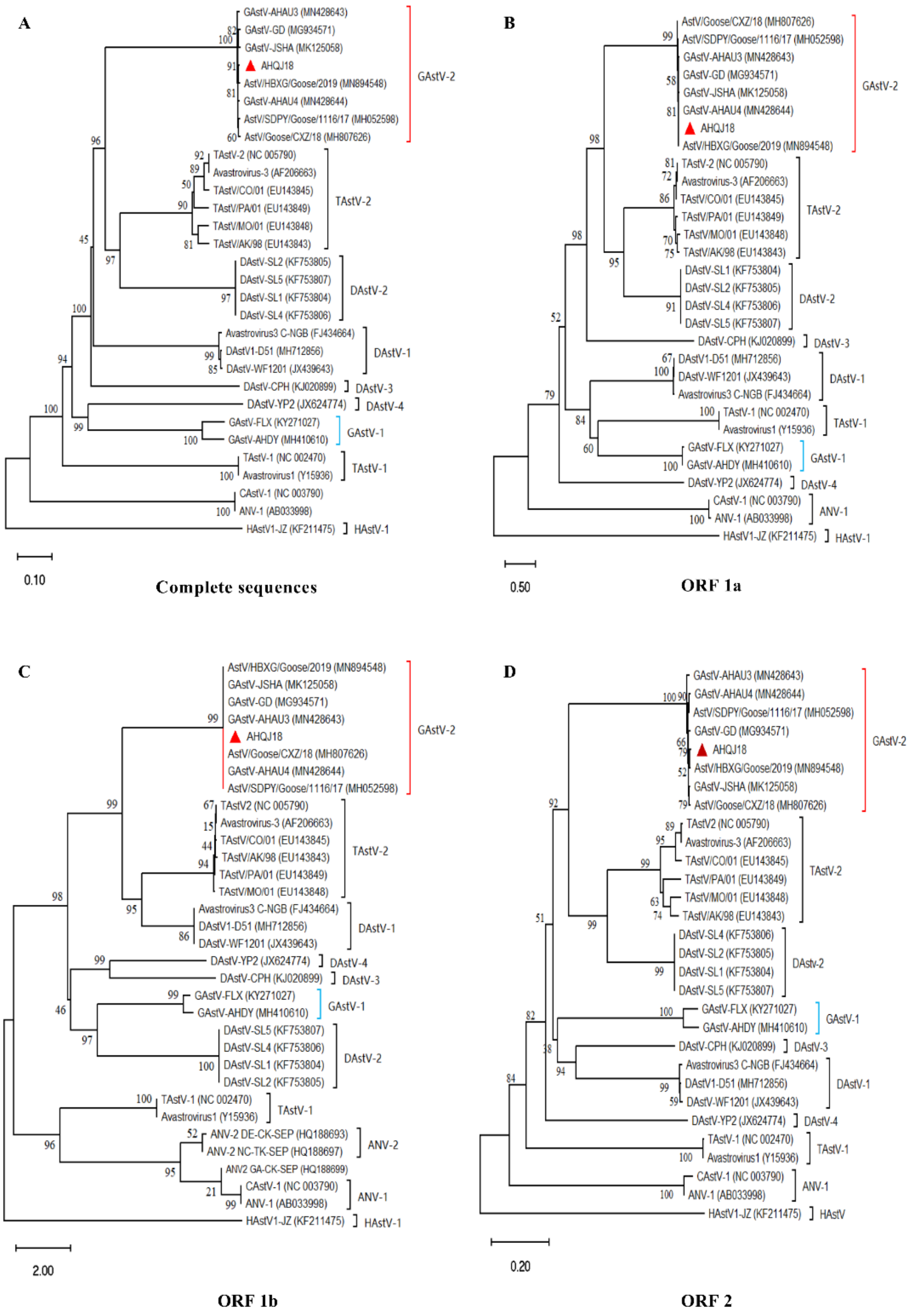

Figure S1. Phylogenetic analysis of GAstV-AHQJ18 strain. (A-D) Phylogenetic analysis of Astrovirus based on the whole genome sequences, ORF1a, ORF1b, and ORF2 was constructed using MEGA 11 software, the Neighbor-joining method with 1,000 bootstrap replicates. The GAstV-AHQJ18 strain is marked with a red triangle.

Table S1. Summary table of amino acid substitutions in the ORF1a, ORF1b and ORF2 region of GAstV-AHQJ18 strain compared with reference GAstV-2 strains.

Accession no.	Strain	ORF1a				ORF2								
		528	580	589	903	208	224	257	464	540	594	614	628	695
MH052598	AstV/SDPY/Goose/1116/17	I	L	H	A	P	T	L	N	Q	S	A	T	T
OP556137	GAstV-AHQJ18	T	L	Y	A	P	A	L	N	L	S	A	T	A
MH807626	AstV/Goose/CXZ/18	T	L	H	S	P	A	L	N	L	G	A	T	A
MN428643	GAstV-AHAU3	I	L	H	A	P	T	L	N	L	S	N	T	A
MG934571	GAstV-GD	I	A	H	A	P	A	L	A	L	N	A	I	A
MK125058	GAstV-JSHA	I	L	H	A	P	A	L	A	L	N	A	T	A
MN428643	GAstV-AHAU4	I	L	H	A	S	A	P	N	L	S	A	T	T

well-recognized mutations compared with classical GAstV-2.

novel mutations compared to classical GAstV-2.

Table S2. A general overview of protective experiments with anti-GAstV specific-IgY in gosling.

Content	Group	Age	Immunization[a]		Infection[b]		Detection[c]
			Dose (mL)	Time	Dose (mL)	Time	Day post inection (DPI)
The minimum effective dose	1	1-day-old	0.3	Day1	0.5	Day2	4, 8, 12
	2	1-day-old	0.6	Day1	0.5	Day2	4, 8, 12
	3	1-day-old	0.9	Day1	0.5	Day2	4, 8, 12
	4	1-day-old	1.2	Day1	0.5	Day2	4, 8, 12
	5	1-day-old	PBS	Day1	0.5	Day2	4, 8, 12
Prophylactic antiviral treatment	6	1-day-old	0.9	Day2	0.5	6 h after immunization	4, 8, 12
	7	1-day-old	0.9	Day2	0.5	12 h after immunization	4, 8, 12
	8	1-day-old	0.9	Day2	0.5	1 d after immunization	4, 8, 12
	9	1-day-old	0.9	Day2	0.5	3 d after immunization	4, 8, 12
	10	1-day-old	0.9	Day2	0.5	5 d after immunization	4, 8, 12
	11	1-day-old	0.9	Day2	0.5	7 d after immunization	4, 8, 12

	12	1-day-old	0.9	Day2	0.5	9 d after immunization	4, 8, 12
	13	1-day-old	PBS 0.9	Day2	0.5	6 h after immunization	4, 8, 12
	14	1-day-old	PBS 0.9	Day2	0.5	12 h after immunization	4, 8, 12
	15	1-day-old	PBS 0.9	Day2	0.5	1 d after immunization	4, 8, 12
	16	1-day-old	PBS 0.9	Day2	0.5	3 d after immunization	4, 8, 12
	17	1-day-old	PBS 0.9	Day2	0.5	5 d after immunization	4, 8, 12
	18	1-day-old	PBS 0.9	Day2	0.5	7 d after immunization	4, 8, 12
	19	1-day-old	PBS 0.9	Day2	0.5	9 d after immunization	4, 8, 12
Post-infection treatment	20	1-day-old	0.9	After infection	0.5	Day 1	4, 8, 12
	21	1-day-old	0.9	1 d after infection	0.5	Day 1	4, 8, 12
	22	1-day-old	0.9	3 d after infection	0.5	Day 1	4, 8, 12
	23	1-day-old	0.9	5 d after infection	0.5	Day 1	4, 8, 12
	24	1-day-old	0.9	none	0.5	Day 1	4, 8, 12

[a]At different days of age or post infection of goslings subcutaneously with the anti-GAstV IgY at a concentration of 2.79mg/ml. [b]Different days of age or post immunization of goslings subcutaneously with 105.84TCID50 GAstV-AHQJ18 strian. [c]4, 8, and 12 days post infection.
